# Shielding of the Geomagnetic Field Alters Actin Assembly and Inhibits Cell Motility in Human Neuroblastoma Cells

**DOI:** 10.1038/srep22624

**Published:** 2016-03-31

**Authors:** Wei-Chuan Mo, Zi-Jian Zhang, Dong-Liang Wang, Ying Liu, Perry F. Bartlett, Rong-Qiao He

**Affiliations:** 1 State Key Laboratory of Brain and Cognitive Science, Institute of Biophysics, Chinese Academy of Sciences, Beijing 100101, China; 2 Queensland Brain Institute, The University of Queensland, Brisbane, Queensland 4072, Australia; 3 Beijing University of Chinese Medicine, Beijing 100029, China; 4 Key Laboratory of Mental Health, Institute of Psychology, Chinese Academy of Sciences, Beijing 100101, China; 5 Alzheimer’s Disease Center, Beijing Institute for Brain Disorders, Capital Medical University, Beijing, 10069, China

## Abstract

Accumulating evidence has shown that absence of the geomagnetic field (GMF), the so-called hypomagnetic field (HMF) environment, alters the biological functions in seemingly non-magnetosensitive cells and organisms, which indicates that the GMF could be sensed by non-iron-rich and non-photo-sensing cells. The underlying mechanisms of the HMF effects on those cells are closely related to their GMF sensation but remain poorly understood so far. Previously, we found that the HMF represses expressions of genes associated with cell migration and cytoskeleton assembly in human neuroblastoma cells (SH-SY5Y cell line). Here, we measured the HMF-induced changes on cell morphology, adhesion, motility and actin cytoskeleton in SH-SY5Y cells. The HMF inhibited cell adhesion and migration accompanied with a reduction in cellular F-actin amount. Moreover, following exposure to the HMF, the number of cell processes was reduced and cells were smaller in size and more round in shape. Furthermore, disordered kinetics of actin assembly *in vitro* were observed during exposure to the HMF, as evidenced by the presence of granule and meshed products. These results indicate that elimination of the GMF affects assembly of the motility-related actin cytoskeleton, and suggest that F-actin is a target of HMF exposure and probably a mediator of GMF sensation.

Migrating animals (for example, the homing pigeon, sea turtle, trout, and butterfly), can use the geomagnetic field (GMF), a weak quasistatic magnetic field (~50 μT)^1^, to guide their navigation in a magnetite and/or photo-sensing-related way^2^. But most species, especially human beings, do not have iron-rich tissues/cells or a cryptochromes (CRYs) dependent photochemical compass^3^,^4^,^5^. Thus, they are commonly believed not able to sense the GMF. Recently, *Drosophila*, which appeared to be non-magnetosensitive, has been trained to exhibit light-sensing-related magnetic-sensing abilities^6^,^7^. Many non-iron-rich and non-photo-sensing cells also showed observable changes after exposure to additional static magnetic fields (SMFs) or GMF attenuation; examples include the proliferation, apoptosis, migration and adhesion of bone and muscle cells^8^,^9^, neuronal cells^10^,^11^,^12^, and cancer cells^13^,^14^,^15^,^16^. However, the mechanisms of their magnetosensation, particularly GMF sensation, remain poorly understood. Assessing the morphological, behavioural, physiological and molecular responses of non-iron-rich and non-photo-sensing cells to a GMF-eliminated environment, called a hypomagnetic field (HMF), will elucidate the mechanisms of their GMF sensation. Moreover, HMF is an environmental factor of outer space^17^; an interest in detailing the response of human beings to the HMF has consequently arisen for long-distance and long-term manned space missions^18^. Investigating the bio-HMF responses of human cells will deepen understanding of both GMF sensation and the potential adverse effects of the HMF.

A body of evidence now supports the conclusion that exposure to the HMF affects embryonic development and brain function. Continuous HMF exposure is reported to result in increased rates of malformation during early embryonic development in newts^19^, frogs^20^, planthoppers^21^, and mice^22^,^23^. A number of studies have reported the negative effects of the HMF on the central nervous system (CNS), which include changed vocal behaviour in birds^24^; perturbed circadian rhythm in birds and humans^25^,^26^,^27^,^28^; and also inhibition of stress-induced analgesia^28^,^30^, decreased noradrenaline release^29^,^31^,^32^, impaired learning and memory^33^,^34^, and weakened visual performance in humans^35^. We previously subjected SH-SY5Y cells to an HMF^13^, and observed, following 2 days of exposure to the HMF, the repression of genes associated with cell migration and assembly of the cytoskeleton in SH-SY5Y cells^12^. In particular, the expression of WASL, an activator of the Arp2/3 complex, which leads to actin nucleation^36^, was down-regulated after 2 days of continuous exposure to the HMF^12^. Cell migration relies on the actin cytoskeleton, as it is responsible for driving the extension of cellular protrusions such as lamellipodia and filopodia at the leading edge of the cell^37^. Considering that cell migration and actin assembly also play important roles in embryonic development and neuroplasticity^38^,^39^ and our findings in SH-SY5Y cells^12^, it is worthwhile studying the effects of the HMF on the motility of SH-SY5Y cells and cytoskeletal assembly. The results will help to elucidate the underlying mechanism of the HMF effect, as well as the role of GMF in these processes.

In the present study, we tested whether the GMF plays a critical role in F-actin-dependent formation of the cytoskeleton and motility of SH-SY5Y cells by using an HMF cell culture system, as described^11^ (Fig. 1a). We found that cells exposed to the HMF showed inhibited capacity for adhesion and migration, a reduction in overall cellular F-actin amount, and a reduction in the density of filopodia. Disordered kinetics of actin assembly *in vitro* was observed during exposure to the HMF, which was provided by a Helmholtz coils system^40^ (Fig. 1b). These results suggest that elimination of the GMF indeed affects the assembly of the motility-related actin cytoskeleton, and that the F-actin cytoskeleton responses to the HMF exposure and probably a mediator in cells’ GMF sensation.

## Results

### HMF exposure reduces cell adhesion and migration

Based on previous evidence suggesting that HMF effects are usually accumulative^17^, we first examined the effect of long-term HMF exposure. A 7-day continuous HMF pre-exposure was applied to SH-SY5Y cells. Cells were passaged every 2 days during the exposure. Suspensions of day 7 HMF and GMF cells (24 h after the third passage) were collected for cell adhesion assays. The adhesive capacities of the pre-exposed cells and the normal control cells to different materials (BSA blank, collagen, fibronectin, 1:1 mixture of collagen and fibronectin, and Matrigel) were compared. HMF pre-exposure significantly reduced (*p* = 0.048) cells’ adhesive capacities to the BSA blank bottom, as compared to the GMF control. This difference was not observed in the groups in which the adhesive coatings were used, indicating that the strength of the adhesive coating materials masked the inhibitory effect of the HMF on cell adhesion (Fig. 2a). Then, we monitored the change in cell adhesion to the blank bottom during the 7-day HMF exposure. The adhesive capacities of cells after 24 h growth at each passage, namely at day 1, day 3, day 5 and day 7 of exposure, were compared between the HMF and GMF groups. The HMF gradually decreased the adhesive capacity, which remained unchanged at day 1 but was significantly reduced at day 3 (p < 0.0001), day 5 (*p* = 0.0007) and day 7 (*p* = 0.0087) (Fig. 2b). Interestingly, the adhesive capacity of the HMF-exposed cells was also repressed at 3 h and 48 h but maintained unchanged at 12 h and 24 h during the first passage (0–48 h) (Fig. 2c).

To verify whether 3 h short-term HMF exposure could directly repress the cell adhesion process, cells were seeded in BSA-coated blank wells and allowed to adhere to the bottom for 3 h in either the HMF or GMF condition. To exclude the potential effects of seeding density on cell adhesion, cells were seeded at 2.0 × 10^4^, 4.0 × 10^4^, or 8.0 × 10^4^ cells/cm^2^. Significantly fewer adhered cells were detected when cells, seeded at 4.0 × 10^4^ cells/cm^2^ (*p* = 0.002) and 8.0 × 10^4^ cells/cm^2^ (*p* = 0.0002), were allowed to adhere in the HMF condition, as compared to the GMF controls (Fig. 2d). These results demonstrate that SH-SY5Y cells can exhibit a quick reduction in cell adhesion capacity in response to short-term (3 h) HMF exposure, and long-term continuous exposure accumulatively reduces the adhesive capacity.

The change in cell adhesion capacity will inevitably affect cell motility. Thus, we firstly measured the vertical migratory capacity by using transwell assay. The results indicated that fewer HMF-exposed cells migrated to the opposite site of the bottom membrane at 4 h (*p* = 0.032) and 24 h (*p* = 0.002) than the GMF controls, and that the migration rates of the HMF-exposed cells were similar to the control group at 2 h and from 6 h to 18 h (Fig. 3a–c). The HMF-induced change in cell migration showed a similar pattern with that in cell adhesion. Next, using a wound-healing assay, we showed that 48 h of continuous exposure to the HMF repressed the horizontal migration (from dense zone to blank zone) of SH-SY5Y cells (p = 0.0003) (Fig. 3d; Fig. S1). The motility of the SH-SY5Y cells was further measured using digital holographic imaging, when cells were allowed to move freely on the horizontal plane (an example image is shown in Fig. S2). The migration speeds of the cells in the HMF group were significantly slower than those in the GMF control group in the first 4 h (2 h, *p* = 0.0007; 4 h, *p* = 0.025), and in the final 24 h (24 h, *p* = 0.010; 36 h, *p* < 0.0001; 48 h, *p* < 0.0001) (Fig. 3e). At all time points analysed, the migration distance of the cells in the HMF group was significantly less than that of the GMF control cells (2 h, *p* < 0.0001; 4 h, *p* < 0.0001; 6 h, *p* = 0.011; 12 h, *p* < 0.0001; 24 h, *p* = 0.0004; 36 h, *p* < 0.0001; 48 h, *p* < 0.0001) (Fig. 2f). The results above confirm that removal of the GMF, and thus exposure to the HMF, results in the inhibition of both cell adhesion and migration. Similar to cell adhesion, SH-SY5Y cells exhibit a quick reduction in cell migration during the first 4 h, and long-term continuous exposure sustains the reduced the migratory capacity.

### HMF-exposed cells exhibit a weakened adhesive morphology

We also recorded the dynamic morphological changes of the cells using holographic imaging. In both conditions (HMF and GMF), cell volume increased during the first 36 h of exposure, and stabilized thereafter (Fig. 4a). From 12 h to 24 h, the cell volume in the GMF group remained stable, whereas that in the HMF continued to increase and was significantly greater than controls at 24 h, indicating that cell growth was enhanced by exposure to the HMF (24 h, *p* = 0.004). But from 36 h to 48 h, cell volumes in the two groups were at the same level. The average thickness of cells exposed to the HMF was greater than those in the GMF group at 4 h and 6 h (Fig. 4b; 4 h, *p* < 0.0001; 6 h, *p* = 0.004), similar with the GMF controls from 12 h to 24 h, and increased significantly again from 36 h to 48 h (36 h, *p* < 0.0001; 48 h, *p* < 0.0001). The area of the cells exposed to the HMF increased from 2 h to 24 h, decreased at 36 h and then was maintained until 48 h (36 h, *p* = 0.007; 48 h, *p* = 0.02) (Fig. 4c). The changes in cell perimeter followed a similar pattern to the changes in cell area (Fig. 4d; 4 h, *p* < 0.0001; 24 h, *p* < 0.0001; 36 h, *p* = 0.003; 48 h, *p* = 0.002). These results suggested that increase in cell thickness from 36 h in HMF was due to a morphological rounding of the cells rather than cell growth.

Two parameters were used to assess cell shape changes: irregularity and eccentricity. Irregularity is how much the circumference of the cell deviates from the circumference of a perfect circle. A value of 0 means the cell is circular and higher value means a longer, more irregular outline. Whereas, eccentricity is how elongated the cell is. A value of 0 corresponds to a circle and the more elongated the cell is the higher the eccentricity value becomes approaching 1. In both groups, the irregularity decreased after 6 h of incubation. As time progressed, the difference between the two groups followed the same trend as the changes in cell area and perimeter length (4 h, *p* = 0.001; 24 h, *p* = 0.008; 36 h, *p* = 0.002; 48 h, *p* < 0.0001) (Fig. 4e). The eccentricity of the cells exposed to the HMF was similar to the controls from 2 h to 36 h, with a significant decrease observed only at 48 h (*p* = 0.001) (Fig. 4f).

Atomic force microscopy showed that, following exposure to the HMF-only environment, cellular processes extended differently compared to those of cells exposed to the GMF (Fig. 5). The protrusion terminals of the cells exposed to the HMF did not extend linearly like those of the controls (Fig. 5b,d), and instead appeared to be retracted (Fig. 5a,c). As shown in Table 1, the average width of the tip of these cells (2.74 ± 0.35 μm) was significantly greater than those exposed to the GMF (1.44 ± 0.22 μm; p = 0.035).

Furthermore, we compared the number of cell processes (N_cp_) extended by cells exposed in the HMF environment to those exposed to the GMF. Cells were classified according to N_cp_, as displayed in Fig. 5e,g–j. The distribution of N_cp_ types in the HMF and GMF cell populations was plotted every 12 h for 48 h (Fig. 5f), and only at 12 h was the distribution of N_cp_ types found to be unaffected by the HMF. At this time point, most of the cells (~80%) displayed multiple processes (N_cp_ ≥ 3). From 24 h to 36 h, SH-SY5Y cells were in the logarithmic growth phase^12^, and the distribution of the N_cp_ types in the cells exposed to the HMF were significantly different from the controls (Chi square test; 24 h, *p* < 0.0001; 36 h, *p* < 0.0001; 48 h, *p* < 0.0001) (Fig. 5f). In the cell populations exposed to the HMF, fewer multiple-process cells (N_cp_ ≥ 3) (24 h, 45.7 ± 0.5%, p = 0.026; 36 h, 53.5 ± 1.3%, p = 0.043; 48 h, 19.2 ± 5.2%, p = 0.022) were observed (GMF: 24 h, 61.6 ± 4.5%; 36 h, 63.1 ± 3.0%; 48 h, 45.3 ± 4.9%). At 24 h, the percentage of null-process (N_cp_ = 0; 10.4 ± 0.4%, p = 0.027), mono-process (N_cp_ = 1; 15.9 ± 0.8%, p = 0.048) and double-process (N_cp_ = 2; 28.0 ± 1.0%, p = 0.036) cells was higher in cells exposed to the HMF, when compared to those incubated in the GMF environment (N_cp_ = 0, 7.2 ± 0.9%; N_cp_ = 1, 9.2 ± 2.2%; N_cp_ = 2, 22.0 ± 1.7%). At 36 h, the percentage of mono-process cells in the HMF (11.9 ± 0.8%, p = 0.0066) was significantly greater than in the GMF (6.6 ± 0.7%). At 48 h, the percentage of multiple-process cells dramatically decreased in cells exposed to the HMF and these numbers remained lower than those observed in the GMF. At this same time point, a greater number of mono-process cells was found in the population exposed to the HMF (24.2 ± 1.1%, p = 0.028; GMF: 12.9 ± 3.1%). Overall, our results show that fewer multiple-process cells were present after exposure to the HMF.

Together, our results indicate that, in accordance with the effect of the HMF on cell motility, the effect of the HMF on cell morphology is also time-dependent. Short-term morphological changes were recorded during the 0–6 h HMF exposure and cells maintained in a less attached morphology, thicker, smaller and rounder in shape, during 24–48 h HMF exposure. Remarkably, the HMF induced cell tip retraction and reduced cell process number. As the outgrowth of cell processes relies on F-actin filamentation^39^, the results above suggest that the HMF may affect the regulation of the F-actin cytoskeleton.

### HMF inhibits actin filamentation

After 48 h incubation in the HMF environment, a greater number of retracted and blunt cell protrusions were observed by F-actin staining, as well as fewer F-actin filopodia at the cell edge, when compared to cells incubated in the GMF (Fig. 6a,b). The amount of F-actin present in cells was significantly reduced after 48 h exposure to the HMF (Fig. 6c; *p* = 0.007), and fewer F-actin fibres were located at the tip of cell protrusions (Fig. 6d,e). The thickness of the F-actin fibres at the tip of the protrusions was similar in both groups (Fig. 6f). To trace the formation of the F-actin filopodia, the density of phalloidin-stained actin fibres near the cell edge was measured at 12 h intervals (Fig. 6g–n). The density of F-actin in the cells exposed to the HMF was not significantly different to the GMF controls from 12 h to 36 h, but was significantly reduced at 48 h (*p* < 0.0001) (Fig. 6o). We next investigated the HMF-responding gene WASL^11^, which is involved in the regulation of actin filamentation^36^. We measured the transcriptional expression of WASL during exposure to either the HMF or GMF at 6 h intervals for 48 h (Fig. 6p). At 48 h, the expression of WASL in the HMF-exposed cells was significantly lower than the GMF control (*p* = 0.019) as reported^14^, but was up-regulated at 6, 12 and 30 h (6 h, *p* = 0.045; 12 h, *p* = 0.011; 30 h, *p* = 0.020), and down-regulated at 18 h (18 h, *p* = 0.036), showing a dynamic expression pattern similar to the genes related to cell proliferation^12^. Considering the “phase-shift” of cell cycle progression in the HMF^11^, the increase in WASL expression might result from the de-synchronization of cell proliferation between the HMF-exposed and GMF-control cells.

### HMF alters the kinetics of actin assembly *in vitro*

We evaluated the effect of the HMF on actin assembly by using the cell-free G-actin *in vitro* self-assembly system. In samples exposed to the GMF, no aggregation of G-actin was observed (Fig. 7b). But in the HMF group, G-actin assembled into fibres and aggregated into granules (diameter, 105.3 ± 23.5 nm) (Fig. 7a,a′). The fibril bundles produced in the HMF were thicker (24.3 ± 10.3 nm, *p* = 0.041) than those produced in the GMF (18.2 ± 5.1 nm) (Fig. 7c). As determined by spectrofluorometry, the kinetics of the assembly reactions were markedly changed by exposure to the HMF. In the GMF, the fluorescence intensity of the assembly mixture increased dramatically before reaching completion at about 60 min (Fig. 7d). The reaction in the GMF was maintained at a constant speed from 0–60 min (K_1G_′ = 14.28 ± 2.79 × 10^−4^·s^−1^; Fig. 7), and was typically a single-phase reaction. However, the changes in fluorescence intensity showed a different kinetic pattern after exposure to the HMF. The fluorescence change in the HMF was a double-phase procedure, containing a fast phase (0–20 min) and a slow phase (20–60 min), with first order rate constants of K_1H_ = 8.67 ± 0.99 × 10^−4^·s^−1^ and K_2H_ = 2.84 ± 0.69 × 10^−4^·s^−1^ (*p* = 0.0017), respectively (Fig. 8; Table 2). The initial assembly constants in the GMF were about 2- and 5-fold greater than the fast and slow phasic constants in HMF, respectively (Fig. 8; Table 2). Interestingly, the increase in fluorescence intensity continued after 60 min in the HMF, indicating the HMF-exposed G-actin was still under active assembly when the GMF control samples had completed the filamentation process. The results suggest that *in vitro* actin self-assembly is sensitive to the HMF.

## Discussion

To date, there has been limited investigation concerning the effect of the GMF on cell adhesion and migration. Our results provide evidence that the GMF is necessary for the maintenance of normal cell morphology, adhesion and motility. Compared to cells incubated in the GMF, cells exposed to the HMF appeared smaller, thicker and rounder, and produced fewer cell processes, displaying retracted morphologies. Following 2 days of exposure to the HMF, both the adhesion and motility of SH-SY5Y cells significantly decreased. The amount of F-actin in cells and the density of F-actin at the cell border also decreased. Increased F-actin containing filopodia formation has been shown to promote migration^41^. Therefore, the HMF-induced reduction in filopodia would contribute to the inhibitory effect of the HMF on cell adhesion and motility. The *in vitro* actin assembly was also found to be disordered in the HMF, both in the reaction rates and products. Therefore, it is probable that GMF elimination acts as an inhibitory extracellular factor on cell motility and morphology by interfering with the F-actin cytoskeleton.

The response of actin assembly to the HMF provides novel insights into the molecular mechanisms underlying the effect of the HMF. Alterations in the regulation of actin assembly results in remarkable changes in the overall architecture of the actin cytoskeleton and, subsequently, the structure of cellular protrusions^42^,^43^,^44^. We confirmed that the protein content of β-actin in the HMF-exposed cells was identical to the GMF controls (Fig. S3); therefore, it is likely that the actin assembly process, rather than actin expression, was disrupted in the HMF. Our *in vitro* experiments showed that exposure to the HMF resulted in disordered actin assembly under a cell-free system, suggesting actin protein may act as a primary responder to the HMF *in vivo*. Exposure to the HMF results in a double-phase rather than a single-phase reaction, which is seen in response to the GMF. Besides the reduced actin assembly rate following HMF exposure, the assembly reaction exhibited an abnormal increase in fluorescence after 60 min, indicating the generation of new products. Our electron microscopy data confirm that, in addition to actin fibrils, aggregated G-actin granules were found in the HMF endproducts. The F-actin bundles produced during exposure to the HMF were thicker than those formed in the GMF, suggesting that the cross-linking between individual F-actin filaments was enhanced in response to the HMF. The energy trade-off between filament twisting and cross-linker binding within an F-actin bundle is suggested to be a fundamental mechanism by which cells can precisely adjust bundle size and strength^45^. The maturation of the actin filament network is sensitive to mechanical stimulation depending on the environmental mechanical tensions^46^,^47^,^48^. The disordered *in vitro* actin self-assembly, degraded cell morphology and motility, and altered WASL expression in response to the HMF suggest that the intracellular F-actin cross-linker system would also be perturbed under the HMF condition. As the *in vitro* system we used was non-enzymatic, it is possible that the conformation of actin protein reacts directly to the exposure of HMF. Furthermore, investigations in plants have revealed that the magneto-sensing gene CRY could be involved in triggering the blue-light induced orchestrated changes of the actin cytoskeleton^49^,^50^. Thus, the interaction between CRYs and the actin cytoskeleton warrants further investigation to clarify the molecular components and their levels in magnetic signal transduction.

On the aspect of the magnetic field conditions, the HMF-simulation set-ups used in our experiments attenuated the background GMF but did not completely eliminate the GMF’s static and alternating components (SMF and AMF), as shown in Table 3. The permalloy chamber attenuated both the SMF and AMF component of the GMF control condition. The residual AMF in the chamber (12.0 ± 0.0 nT at 50 Hz) was ~2% of the weak power line generated AMF in the GMF control (575.7 ± 29.1 nT at 50 Hz). It has been shown that power frequency MF exposure acutely affects the migration/motility-related actin cytoskeleton reorganization in human amniotic epithelial cells^51^. It is proposed that power frequency MF-induced changes in the cytoskeleton are due to the presence of field-cell interaction sites at the plasma membrane level^51^,^52^,^53^, which may subsequently trigger a regulatory system that attempts to restore the HMF-disordered actin assembly. Therefore, the field-cell interaction is likely reduced by the HMF condition and would lead to a weakened restoration on the disordered actin assembly. Baek *et al.* have reported that cancellation of the AMF component of the GMF does not change the viability and the expression of cell cycle related genes in fibroblast cells and embryonic stem cells but does inhibit epigenetic reprogramming during induced pluripotent stem cell generation^54^. The HMF would also affect the actin-cytoskeleton system in an epigenetic way in terms of the weakened AMF in our HMF cell culture system. The Helmholtz coils system (HCS) compensated only the SMF component of the local GMF with direct current and the AMF was similar inside (12.8 ± 1.0 nT at 50 Hz) and outside (14.0 ± 1.0 nT at 50 Hz) the HCS. Actin assembly alterations were observed in the two HMF conditions generated by the shielding chamber and the HCS. Therefore, it is reasonable to infer that the observed biological effect of the HMF condition is largely attributable to the attenuation of the SMF component of the GMF. Magnetic effect and electric effect are commonly assumed as an integrated effect, which cannot be thoroughly separated. In non-iron-rich and non-photo-sensing cells, electromagnetic properties, especially the neural electrophysiological processes in neurons, are another potential target of the applied AMFs and SMFs. Attenuation of the GMF will proportionally reduce the impact of the GMF on electric signal transduction in the nervous system and lead to the sensation of the change in the environmental magnetic field. The changes in cellular behaviour and molecular regulation under the HMF condition are possibly the downstream effects of the bio-electromagnetic interaction, which result from the weakening of both the SMF and AMF components of the GMF.

Although the inhibitory effect of the HMF on cytoskeleton was obtained from SH-SY5Y cell, a tumour cell line here, the response of cytoskeleton to HMF was also reported in mice primary embryo cultures^22^ and *Xenopus* embryonic development^20^, indicating the HMF-induced interference on cytoskeleton could be found both in normal cells and tumour cells. Therefore, the HMF not only could alter embryonic development but also would affect tumour progression. Abundant filopodia have been described as a characteristic of invasive carcinoma cells^55^. Cell adhesion, cytoskeletal organization and motility plays essential roles in tumour progression. Cancer therapeutics are usually designed to target adhesion receptors^56^. HMF-exposed SH-SY5Y cells showed a reduction in F-actin content, cell adhesion and motility, suggesting the HMF a potential choice to prevent tumour progression for the clinical therapy. However, the proliferation of SH-SY5Y is accelerated in the HMF^11^. Although inhibitory effect of the HMF on several cancer cell lines were reported^14^,^57^, the risk of accelerating certain tumour growth in the HMF should not be ignored in both the future clinical application and navigation in the deep space.

Previous experiments using short-term exposure to the HMF showed marked changes in oxidative stress levels^58^,^59^ and sensitivity to environmental stimuli^11^,^17^, suggesting that the HMF may trigger a quick stress response. In our study, the changes in cell motility, cell morphology and WASL expression under the HMF condition also showed an acute response during the initial period of exposure (0–6 h) but followed with responses generally slowing and stabilizing during the final 12 hours of incubation (36–48 h). The observed time-dependent cellular response to the HMF is consistent with our previous findings. Most of the HMF-responding genes exhibit an early–quick (0–12 h) and late-sustained expression pattern in the HMF^12^. This indicates that future experiments should carefully consider the duration of exposure on testing the effect of the HMF. However, the cellular changes (cell adhesion, migration and morphology) observed during the first 6 h of exposure to the HMF were, in most cases, restored to control levels over the next 12 h (12–24 h), indicating that the bio-system can compensate for the short-term actions of the HMF. But the inhibitory effects of cell adhesion and migration, cell protrusion growth and actin assembly sustained after long-term HMF exposures, confirming that the action of the HMF is potentiated over time^17^. Considering the roles of cell motility and actin cytoskeleton in maintaining cell viability and the function of the CNS and the immune system, the “short-term” reversible and “long-term” accumulative properties of the HMF responses suggest that astronauts during the deep space mission would potentially experience a stressful stage at the initial HMF exposure period and suffer from accumulated HMF-induced alteration. The risk of both effects should not be ignored in the health care of the astronauts.

From the results of the present study, we can conclude that GMF shielding is a stress factor causing significant morphological (cell protrusion, process number and morphology) and functional changes (adhesion and migration) in SH-SY5Y cells. GMF shielding acts as a negative regulator of cell activity and actin assembly. The regulation system of actin assembly can response to the HMF exposure and is likely to be a mediator in cell’s GMF-sensation. These findings suggest that the HMF would potentially be used to regulate actin-related activities and functions, which are also clues to foresee the risks of HMF exposure in deep space to astronauts,

## Methods

### The HMF Conditions

**Figure 1:**
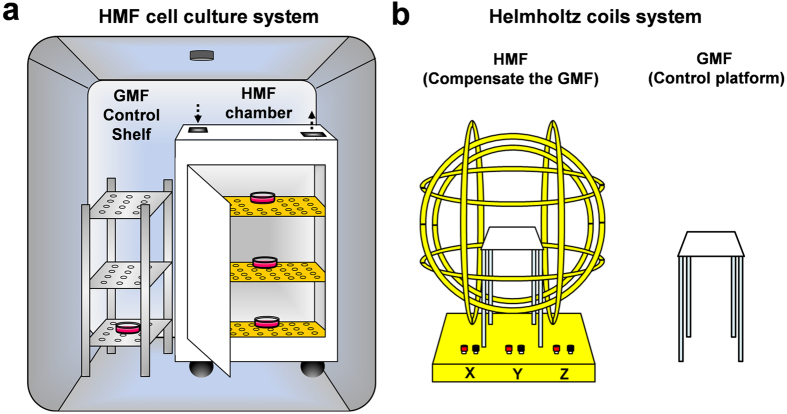
Simulation of the HMFs. (**a**) A permalloy magnetic shielding chamber is settled in a cell incubator and provides a <200 nT HMF condition for cell culture. The GMF control cells are placed on the bottom layer of a steel shelf aside the permalloy chamber. (**b**) A three-axis Helmholtz coils system compensates the GMF at the center of the system and provides a <500 nT HMF condition for *in vitro* actin assembly. The GMF control samples are placed on a platform outside the coils system.

**Figure 2:**
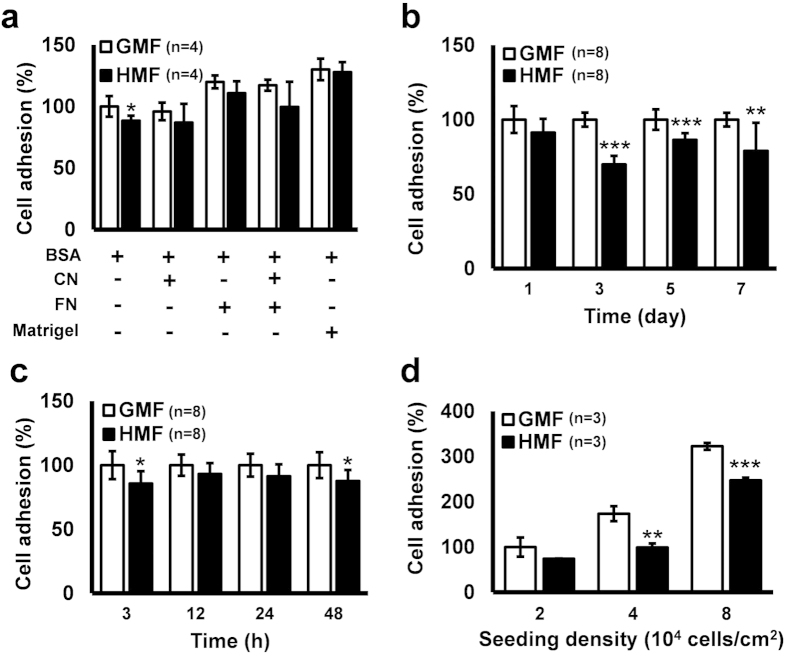
Decreased cell adhesion in the HMF. (**a**) After 7-day HMF or GMF pre-incubation, SH-SY5Y cells were allowed to adhere to wells pre-coated with human Collagen IV (CN; 40 μg/ml), human Fibronectin (FN; 40 μg/ml), 1:1 FN (20 μg/ml) and CN (20 μg/ml) mixture, 1:100 diluted Matrigel, or blank wells pre-coated with BSA (50 mg/ml). The cell adhesion assay showed that, in the blank wells, the percentage of adhesion of the cells exposed to the HMF was significantly lower than the GMF control. (**b**) The HMF exposure gradually reduced cell adhesive capacity. The adhesive capacities of day 1, day 3, day 5 and day 7 HMF-exposed cells were significantly reduced. (**c**) During 2-day HMF incubation, the adhesive capacity was reduced at 3 h and 48 h but maintained unchanged at 12 h and 24 h. (**d**) Cells from GMF condition were seeded in BSA coated blank wells at different densities and transferred to the HMF or GMF for a 3 h, short-term exposure. The adhesion ratios of the cells exposed to the HMF were significantly lower than the GMF controls at 4.0 × 10^4^ cells/cm^2^ and 8.0 × 10^4^ cells/cm^2^. n is the number of separate experiments. Data are shown as mean ± s.d. The *P* values were calculated using one-way ANOVA. *P < 0.05, **P < 0.01, ***P < 0.001.

**Figure 3:**
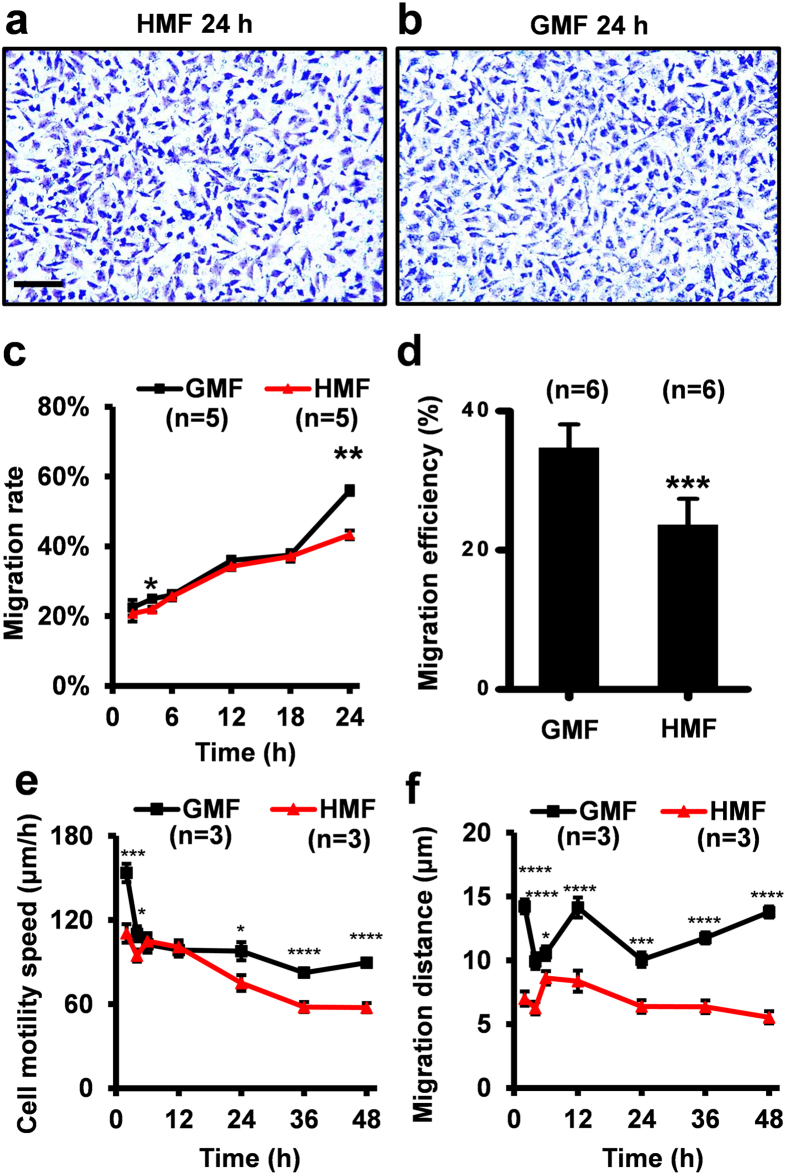
Decreased cell migration in the HMF. Representative crystal violet staining of the migrated cells on the transwell inserts in the HMF (**a**) and GMF (**b**) at 24 h. (**c**) The migration rate of the HMF-exposed cells was significantly slower than the GMF controls at 4 h and 24 h. (**d**) The migration efficiency of the cells incubated in the HMF-only environment was significantly less than the controls in the wound-healing assay. (**e**) The motility speed of the cells exposed to the HMF was significantly slower than the GMF control at 2–4 h and 24–48 h. (**f**) The migration distance of the HMF-exposed cells was smaller than the GMF control during the 48 h incubation period. Data from separate experiments (n = 5 in **c**; n = 6 in b; n = 3 in **e**,**f**) are shown as mean ± s.e.m. The *P* values were calculated using a one-way ANOVA. *P < 0.05, **P < 0.01, ***P < 0.001, ****P < 0.0001.

**Figure 4:**
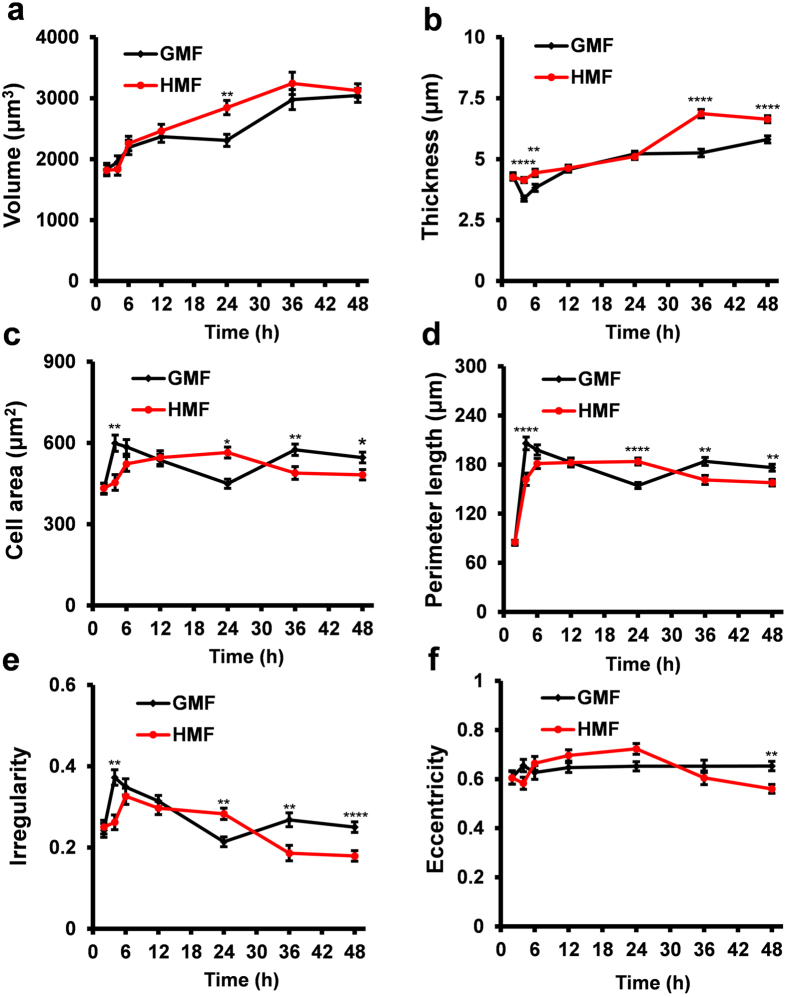
Morphological parameters of the HMF-exposed cells. Cell morphology parameters of SH-SY5Y cells, including cell volume (**a**), thickness (**b**), area (**c**), perimeter length (**d**), irregularity (**e**) and eccentricity (**f**) were measured using holographic imaging at 2, 4, 6, 12, 24, 36 and 48 h during exposure to the HMF or GMF. The volume of the HMF-exposed cell was larger than the GMF control at 24 h (**a**). The HMF-exposed cells were thicker than the controls at 4–6 h and 36–48 h (**b**). The HMF-exposed cells have larger cell areas and perimeter lengths at 24 h and smaller at 4 h and 36–48 h, as compared to the controls (**c**,**d**). The irregularity of the HMF-exposed cells increased at 24 h but decreased at 4 h and 36–48 h when compared to the control (**e**). The eccentricity of the HMF-exposed cells was decreased at 48 h (**f**). Data from three independent experiments are shown as mean ± s.e.m. The *P* values were calculated using a one-way ANOVA. *P < 0.05, **P < 0.01, ****P < 0.0001.

A permalloy magnetic shielding chamber was used to create the HMF environment (SMF, <200 nT; AMF, 12.0 ± 0.0 nT at 50 Hz) for cell culture (Fig. 1a; Fig. S4a), as described in earlier studies^11^. The control cells were placed on the bottom layer of a stainless steel shelf (SMF, 15.1 ± 2.2 μT; AMF, 575.7 ± 29.1 nT at 50 Hz) outside the shielding chamber (Tab. 3; Fig. S4b).

A 3-axis HCS (Fig. 1b) was used to provide the HMF condition for *in vitro* actin assembly reaction, as described previously^40^. At the HCS center, the residue SMF was 470.0 ± 13.7 nT at the HMF mode (Tab. 3; Fig. S5); the AMF was 12.8 ± 1.0 nT at 50 Hz. The control cells were incubated on a plastic platform of the same height of the HCS center outside the HCS and separated by a distance of 20 cm (SMF, 52.5 ± 0.4 μT; AMF, 14.0 ± 1.0 nT at 50 Hz).

The SMF was measured using a 3-axis fluxgate magnetometer. The AMF was measured using a CCG-1000 induction alternative magnetometer.

### Cell Culture

SH-SY5Y cells were maintained in DMEM (High D-glucose) supplemented with 10% (v/v) fetal bovine serum (FBS), 100 unit/ml penicillin and 100 μg/ml streptomycin. They were kept as a monolayer in petri dishes at 37 °C, 5.0% CO_2_ concentration and >95% relative humidity.

### Cell Adhesion Assay

The cell adhesion assay was conducted by following a previously-established protocol from Professor Xia Chen’s laboratory website (Department of Bioengineering and Therapeutic Sciences, University of California San Francisco, USA; http://dbts.ucsf.edu/chen/Protocols.htm). The wells were treated with bovine serum album (BSA, 50 mg/ml) prior to the coating procedure. Wells treated with BSA alone were called the “blank group”. Human Fibronectin (FN, 40 μg/ml; Sigma-Aldrich, St. Louis, MO, USA), human Collagen IV (CN, 40 μg/ml; Sigma-Aldrich, St. Louis, MO, USA), 1:1 mixture of FN (20 μg/ml) and CN (20 μg/ml), and 1:100 dilution of Matrigel (Bio-Rad Laboratories, Hercules, CA, USA) solutions were applied to coat individual wells. All solutions were prepared in DMEM without FBS. Cells were exposed to the HMF or GMF conditions before the cell adhesion assay. For long-term exposure, cells were continuously passaged in a two-day interval and seeded in 100 mm petri dishes at a density of 2.0 × 10^4^ cells/cm^2^ for each passage. During the first 2-day exposure, cell adhesion was measured at 3 h, 12 h, 24 h and 48 h. In order to exclude the potential interference of the pro-proliferation effect of the HMF^11^, cell adhesion was measured 24 h after the passage (day 3, day 5 and day 7) (See cell adhesion assay results of day 2, day 4 and day 6 in Fig. S6). At each measuring time point, cells were reseeded at 9.0 × 10^4^ cells/cm^2^ in the prepared plates for cell adhesion assay. After 1 h incubation at 37 °C in the GMF, the plates were subjected to a defined dislodgement shear force by centrifugation (200 rpm, 2 min). Crystal violet staining was used to visualize the number of the remaining adherent cells, as described previously^11^. The cell adhesion rates were standardized to the blank GMF group.

For the short-term exposure to the HMF, cells were seeded at different densities (2.0 × 10^4^, 4.0 × 10^4^, and 8.0 × 10^4^ cells/cm^2^) in 12-well plates and immediately transferred into either the HMF or GMF. BSA pre-treatment, dislodgement centrifugation and crystal violet staining were performed as described above. The cell adhesion rates were standardized to the blank GMF group with the lowest seeding density.

### Transwell assay

The transwell assay (24-well, 8.0 μm polycarbonate membrane; Corning, USA) was used to measure the cell migratory ability. Given the pro-proliferative effect of HMF exposure^11^ may interfere with cell migration, cells were stimulated to migrate in DMEM solution containing a low concentration of FBS (1% FBS). Serum-starved cells were seeded at 5 × 10^4^ cells/insert and immediately transferred into the HMF or GMF. After exposure, cells were fixed with 4% paraformaldehyde for 10 min at room temperature (RT) and stained with crystal violet. The number of cells that had migrated (N_m_) to the outer edge of the insert was counted using a phase contract microscope (200 × magnification). The migration rate was calculated as: 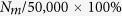.

### Wound healing assay

SH-SY5Y cells were seeded into a 6-well plate in DMEM with 10% FBS and grown to a monolayer. A ‘wound lesion’ was made in each well using a pipette tip. In order to repress cell proliferation, cells were incubated in DMEM with 0.5% BSA. Cells were then transferred to the HMF or GMF incubation condition. The lesion width at 0 h (D_0_) and after 2 days incubation (d) was measured. The migration efficiency was calculated as: 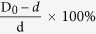.

### Digital Holographic Imaging

Cell motility (speed and distance) was measured using three-dimensional holographic imaging (HoloMonitor M4; Phase Holographic Imaging, Lund, Sweden)^60^. Cells were seeded at 2.5 × 10^3^ cells/cm^2^ in T25 tissue culture flask (Corning, Steuben county, NY, USA) and maintained at DMEM with 1% FBS in either the GMF or HMF. In each trial, the flasks were transferred to the holographic microscope at the observation time points and digital photos were taken randomly every 30 s for 30 min. The frame at the 15^th^ min of each imaging period was used for a cell morphology assay. The cell morphology parameters (cell area, volume, thickness, perimeter length, eccentricity and irregularity) were measured using Hstudio M3 2.6 tracker (Phase Holographic Imaging, Lund, Sweden). At each time point, at least 83 individual cells in total were measured for the motility parameters, and at least 110 individual cells for the morphological parameters.

### Atomic Force Microscopy (AFM)

Cells were seeded in 60 mm petri dishes at 2.5 × 10^3^ cells/cm^2^ and maintained in DMEM with 10% FBS. After 48 h incubation, cells were fixed using 4% paraformaldehyde. The GMF control cells and the multiple-process (N_cp_ ≥ 3) HMF-exposed cells were scanned using AFM (NanoscopeIIIa multi-mode picoforce, VEECO, USA) (80 × 80 μm^2^ observation window). The morphology parameters of the cell front protrusions were measured.

### Quantification of Cell Process Numbers (N_cp_)

**Figure 5:**
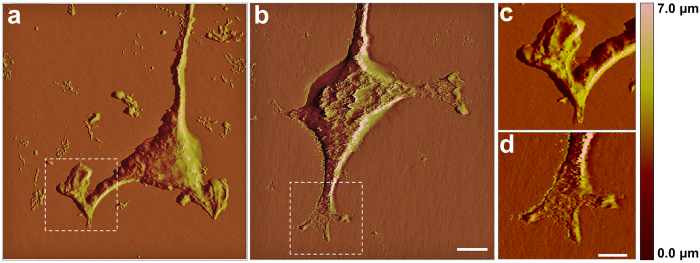
Decreased cell process numbers in the HMF. (**a–d**) Representative atomic force microscope scanning photos show cell protrusion structure of the cells exposed to the HMF (**a**) and GMF (**b**) after 48 h incubation. An enlarged view of the cell front protrusions of the cells incubated in the HMF (**c**) or GMF (**d**). The cell front protrusions of the HMF-exposed cells appeared retracted (**c**), whereas those of the GMF control cells were out-stretched (**d**). (**e–j**) The number of cell processes (N_cp_) was determined at 12 h intervals when cells were maintained under DMEM with 10% FBS. (**e**) A representative image of the HMF-exposed cells fixed at 24 h. The arrows indicate cell types with different CP numbers (N_cp_). Blue, N_cp_ = 0; Green, N_cp_ = 1; Yellow, N_cp_ = 2; Red, N_cp_ ≥ 3. (**f**) The distribution of cell types with different N_cp_ in the HMF and GMF conditions from 12 h to 48 h. The distributions of N_cp_ were different between the HMF and GMF groups at 24, 36 and 48 h; specifically, fewer multiple-process cells (Ncp ≥ 3) were counted in the HMF. Data from three independent experiments, are shown as mean ± s.e.m. The *P* values were calculated using a Chi-square test. ****P < 0.0001. Representative images of N_cp_ cell types were shown as: N_cp_ = 0 (**g**), N_cp_ = 1 (**h**), N_cp_ = 2 (**i**) and N_cp_ ≥ 3 (**j**).

**Figure 6:**
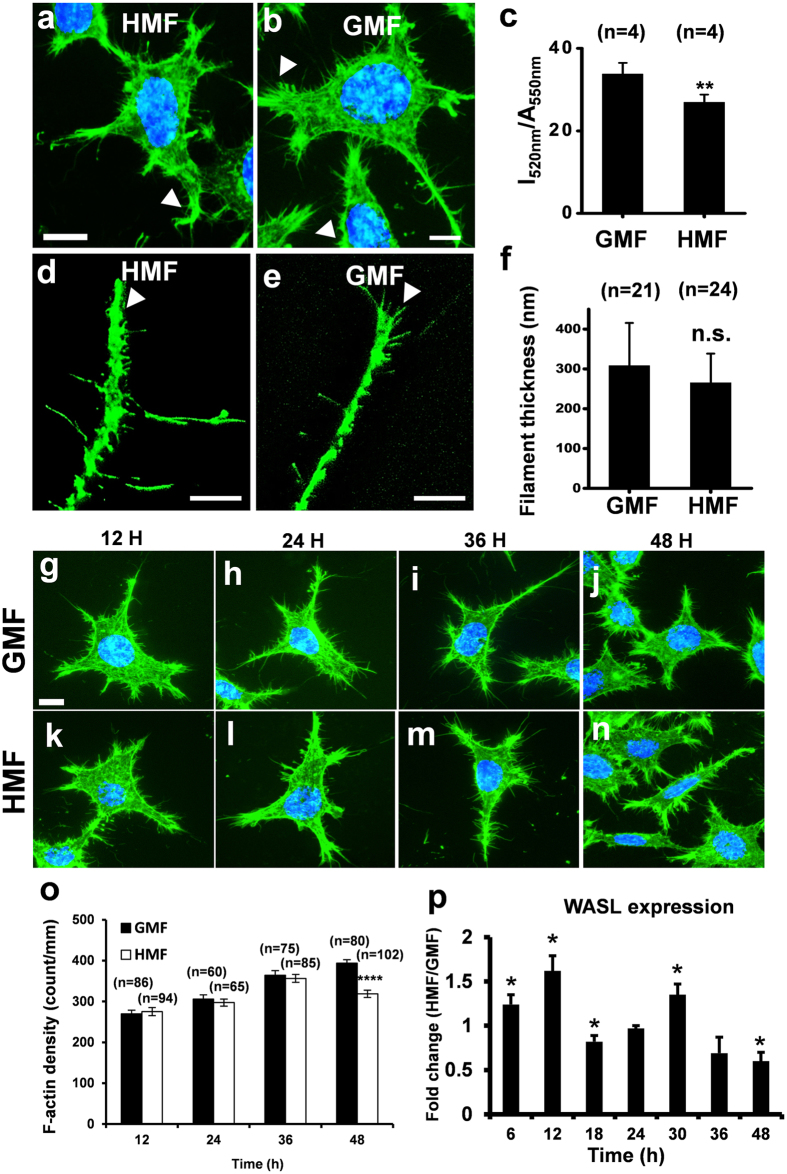
Reduced cellular F-actin in HMF-exposed cells. Representative images of cells exposed to either the HMF (**a**) or GMF (**b**) after 2 days. Cells are stained with FITC-phalloidin (green). The nuclei were stained with Hoechst (blue). Typical cell front protrusions are indicated using white arrowheads. (**c**) Quantification of the cellular F-actin showed that 2 days of HMF exposure results in cells with significantly less F-actin. After 2 days of exposure to the HMF (**d**) and GMF (**e**) fewer F-actin fibers were located at the tip of cell protrusion of the HMF-exposed cells than the GMF control. (**f**) Thicknesses of F-actin fibers of cells in the GMF and HMF conditions were similar. Representative photos of FITC-phalloidin-stained SH-SY5Y cells in the GMF (**g–j**) and HMF (**k–n**) at 12 h (**a,k**), 24 h (**h,f**), 36 h (**i,m**) and 48 h (**j,n**). (**o**) The F-actin densities of the HMF-exposed cells was not significantly different to the GMF controls from 12 h to 36 h but decreased significantly at 48 h. (**p**) The dynamic relative expression levels of WASL (HMF/GMF) during the 48 h exposure period. The transcriptional expression of WASL of the HMF cells was lower than the GMF control at 18 h and 48 h but higher from 6–12 h and at 30 h. Data in (**c**) were from independent experiments (n = 4), and are shown as mean ± s.e.m. Data in (**f**) were from randomly-selected individual fibers from three independent experiments (n is the number of actin fibers), and are shown as mean ± s.d. Data in (**o**) were from randomly selected cells from three independent experiments (n is the number of cells), and shown as mean ± s.e.m. Data in (**p**) were from three independent experiments at each sampling time point, and shown as mean ± s.e.m. The *P* values were calculated using a one-way ANOVA. *P < 0.05, **P < 0.01, ****P < 0.0001.

**Figure 7:**
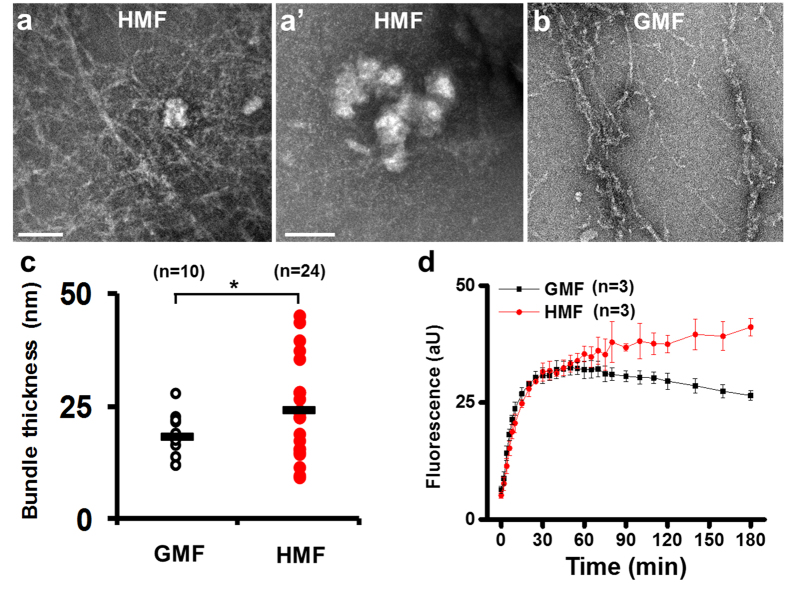
Disordered *in vitro* actin self-assembly in the HMF. Electron microscopy of the end products of actin self-assembly reaction showed that fibers and granules were formed in the HMF (**a**,a′) but only fibers were found in the GMF (**b**). (**c**) Fibrils produced in the HMF were found to be significantly thicker than those produced in the GMF. (**d**) The fluorescence changes of the actin self-assembly mixtures were monitored for 3 h. The reaction kinetics changed remarkably after 60 min. Data in (**c**) were measured from randomly selected individual fibrils (**n**) from three independent experiments. The *P* values were calculated using the Kolmogorov-Smirnov test. *P < 0.05. Data in (**d**) were from three independent experiments and are shown as mean ± s.d.

**Figure 8:**
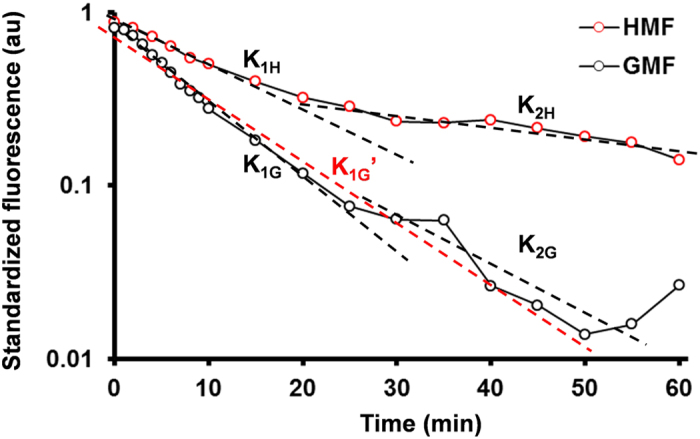
Kinetics of the actin self-assembly reactions. The fluorescence of actin assembly mixtures (0–60 min) is shown using semilogarithmic plotting. The first-order rate constants were determined. The assembly reaction in the HMF was a double-phase process (K_1H_ & K_2H_). The assembly reaction in the GMF was a single-phase process (K_1G′_). The values of first-order rate constants were shown in Table 2.

Cells were maintained under the same conditions as described in the section above, collected every 12 h and fixed using 100% methanol. Photos were taken using a phase contract microscope. Cells were classified according to their N_cp_ (N_cp_ = 0; N_cp_ = 1; N_cp_ = 2; N_cp_ ≥ 3) at randomly selected fields (200×) (Fig. 5C–F). The number of each cell type was counted.

### F-actin Staining and Quantification

The amount of cellular F-actin was quantified as described previously^61^,^62^. Cells were seeded in 35 mm petri dishes at a density of 1.0 × 10^4^ cells/cm^2^ and incubated in DMEM with 10% FBS for 48 h. Cells were stained with 1 μM FITC-phalloidin (Sigma-Aldrich, St. Louis, MO, USA)^63^ The cells, with bound FITC-phalloidin, were then washed with 10 ml 100% methanol on a rocking platform. The fluorescence intensity of the extracted FITC-phalloidin solution was measured using a fluorimeter (Hitachi F-4500, Chiyoda, Tokyo, Japan) at excitation/emission wavelengths of 480/520 nm (I_520nm_). After FITC-phalloidin extraction, crystal violet staining was performed (A_550nm_) to quantify the number of cells remaining in the dishes, as described above. The cellular F-actin amount was determined as: 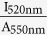.

For F-actin density quantification, cells were grown on coverslips in 35 mm petri dishes (1.0 × 10^4^ cells/cm^2^), and stained with FITC-phalloidin and photographed using a fluorescence microscope (Olympus IX71) with Olympus CCD DP71 (Olympus, Shinjuku, Tokyo, Japan). The number of F-actin fibers (N_F-actin_) around the edge of the cell was counted and cell perimeters (C) were measured. F-actin density was determined as: N_F-actin_/C (count/mm). F-actin fibers at the tip of cell adhesion structure were observed using a Total Internal Reflection Fluorescence (TIRF) microscope (Olympus, Shinjuku, Tokyo, Japan). The thickness of F-actin fibers was also measured. Calculations were made using Image-Pro Plus 6.0.

### qPCR analysis

The gene-specific primers were designed by PrimerBank (http://pga.mgh.harvard.edu/primerbank)^64^ and synthesized commercially. The primers are listed in Table 4. The qPCR samples were run in triplicate on a Rotor gene QPCR cycler (QIAGEN, Valencia, CA, USA) using a TransStart Green qPCR SuperMix UDG kit (TransGen Biotech, Beijing, China). The relative quantitation of gene expression was normalized to *α-tubulin*. The expression of HMF sample genes was normalized to the corresponding GMF control.

### *In vitro* Actin Assembly

Global-actin (G-actin) was freshly purified from rabbit hind leg muscle, as described previously^65^,^66^ and purified monomer actin was conjugated with a fluorescent probe, pyrene (Sigma-Aldrich, St. Louis, MO, USA), as described previously^67^ (Fig. S7). The fluorescence of the reaction mixture at excitation/ emission wavelengths of 285/407 nm (I_407nm_) with a fluorimeter (Hitachi F-4500, Chiyoda, Tokyo, Japan). The kinetics of self-assembly reactions were analyzed by semilogarithmic plotting, as described previously^40^.

### Electron microscopy

The end products of *in vitro* actin assembly were observed with electron microscopy, as described previously^40^. Samples were finally visualized using a JEM-100CX electron microscope (JEOL, Tachikawa, Tokyo, Japan). The thicknesses of fibers and the diameter of granules in the end products were measured using Image-Pro Plus 6.0.

### Statistical Methods

Each experiment was repeated independently at least three times. One-way analysis of variance (ANOVA) was applied for mean comparison. The Chi-square test was applied for the analysis of the distribution of N_cp_ types. The Kolmogorov-Smirnov test was used to compare the assembled actin bundle thickness. A *P* value < 0.05 was considered to be statistically significant.

## Additional Information

**How to cite this article**: Mo, W.-C. *et al.* Shielding of the Geomagnetic Field Alters Actin Assembly and Inhibits Cell Motility in Human Neuroblastoma Cells. *Sci. Rep.* **6**, 22624; doi: 10.1038/srep22624 (2016).

## Supplementary Material

Supplementary Information

## Figures and Tables

**Table 1:** Cell morphology parameters.

Unit (μm)	HMF	GMF
Cell branch Height	1.40 ± 0.11	1.72 ± 0.18
Cell branch Width	4.49 ± 0.49	3.74 ± 0.08
Cell tip Height	0.06 ± 0.02	0.04 ± 0.01
Cell tip Width	2.74 ± 0.35*	1.44 ± 0.22

Data represent the mean ± s.e.m. of at least three independent experiments. *p < 0.05.

**Table 2:** The first order rate constants of fluorescence changes during actin assembly^f^.

Phase	HMF	GMF	
Fast phase (0–20 min)	K_1H_ = 8.67 ± 0.99^a^	K_1G_ = 17.27 ± 2.22**^b^^,^^c^	K_1G_′ = 14.28 ± 2.79$^d^
Slow phase (20–60 min)	K_2H_ = 2.84 ± 0.69##^e^	K_2G_ = 12.00 ± 6.37	/

^a^Data were shown in mean mean ± s.d. from three independent experiments.

^b^K_1H_ < K_1G_; **p = 0.0036, calculated by student’s t-test.

^c^K_1G_ is not significantly different from K_2G_; p = 0.3932, calculated by paired student’s t-test;

^d^Calculated from the GMF data from 0 to 60 min (red dashed line); K_1H_ < K_1G_′; ^$^, p = 0.0224, calculated by student’s t-test.

^e^K_1H_ > K_2H_; ^##^p = 0.0017, calculated by paired student’s t-test.

^f^The rate constants (K) are the absolute values in 10−4·s−1.

**Table 3:** The magnetic field conditions.

	SMF (μT)	AMF (nT at 50 Hz)
Magnetic shielding chamber (HMF)	0.121 ± 0.054	12.0 ± 0.0
Control in the incubator (GMF)	15.1 ± 2.2	575.7 ± 29.1
Helmholtz coils system (HMF)	0.470 ± 0.014	12.8 ± 1.0
Experiment room (GMF)	52.5 ± 0.4	14.0 ± 1.0

Data represent the mean ± s.d. of three independent measurements.

**Table 4:** Primers used for qPCR assay.

Gene	Primer	Sequence (5′ to 3′)	T_m_ (°C)	Length (bp)
α-tubulin	forward	ACCTTAACCGCCTTATTAGCC	52	288
	reverse	CACCACGGTACAACAGGCA		
WASL	forward	GAACGAGTCCCTCTTCACTTTC	50	110
	reverse	CACTGCACTTCTTTGACCACATA		
