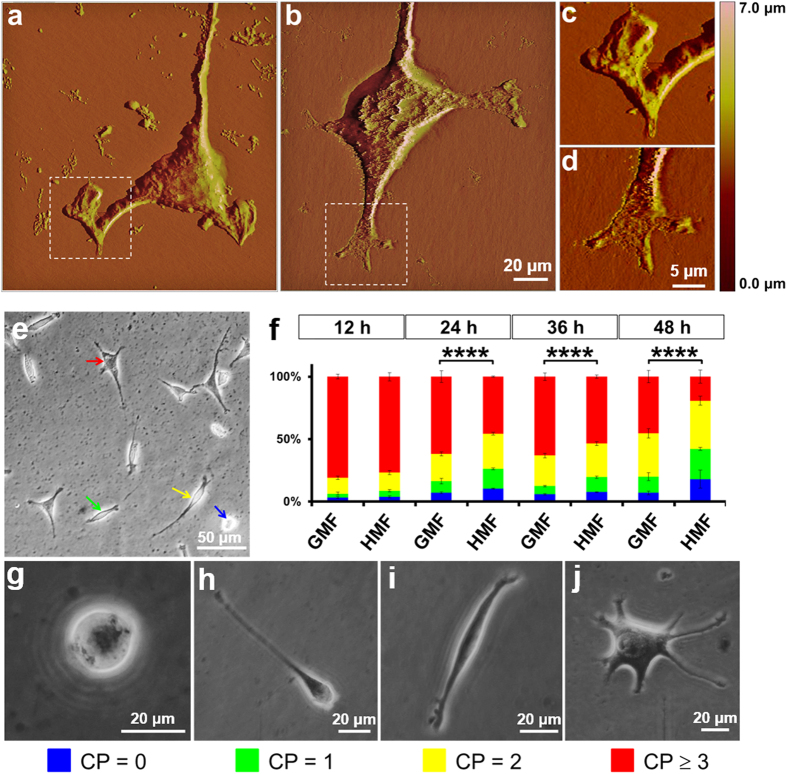# Corrigendum: Shielding of the Geomagnetic Field Alters Actin Assembly and Inhibits Cell Motility in Human Neuroblastoma Cells

**DOI:** 10.1038/srep32055

**Published:** 2016-08-30

**Authors:** Wei-Chuan Mo, Zi-Jian Zhang, Dong-Liang Wang, Ying Liu, Perry F. Bartlett, Rong-Qiao He

Scientific Reports 6: Article number: 2262410.1038/srep22624; published online: 03 31 2016; updated: 08 30 2016

In this Article, Figures 5e-j are omitted. The Figure legend is correct. The correct Figure 5 appears below as Figure 1.

## Figures and Tables

**Figure 1:**