# Supplementary material for: Shielding of the Geomagnetic Field Alters Actin Assembly and Inhibits Cell Motility in Human Neuroblastoma Cells
Source: Sci Rep. 2016 Mar 31;6:22624. doi: 10.1038/srep22624 (PMC4814845; doi:10.1038/srep22624)
Supplement: Supplementary Information [file srep22624-s1.doc]

**Shielding of the Geomagnetic Field Alters Actin Assembly and Inhibits Cell Motility in Human Neuroblastoma Cells.**

Weichuan Mo^1,2^, Zijian Zhang^1,3^, Dongliang Wang^1^, Ying Liu^1^,*, Perry F. Bartlett^2^,*, Rongqiao He^1,4,5^

^1^State Key Laboratory of Brain and Cognitive Science, Institute of Biophysics, Chinese Academy of Sciences, Beijing 100101, China

^2^Queensland Brain Institute, The University of Queensland, Brisbane, Queensland 4072, Australia

^3^ Beijing University of Chinese Medicine, Beijing 100029, China

^4^Key Laboratory of Mental Health, Institute of Psychology, Chinese Academy of Sciences, Beijing 100101, China

^5^Sichuan Medical University, Luzhou, Sichuan 646000, China

***Corresponding authors**: Ying Liu (Email: liuy@moon.ibp.ac.cn, Tel.: +86-10-64875055), Perry Bartlett (Email: [p.bartlett@uq.edu.au](mailto:p.bartlett@uq.edu.au), Tel.: +61-7-33466403)

**Supplementary Information**

**
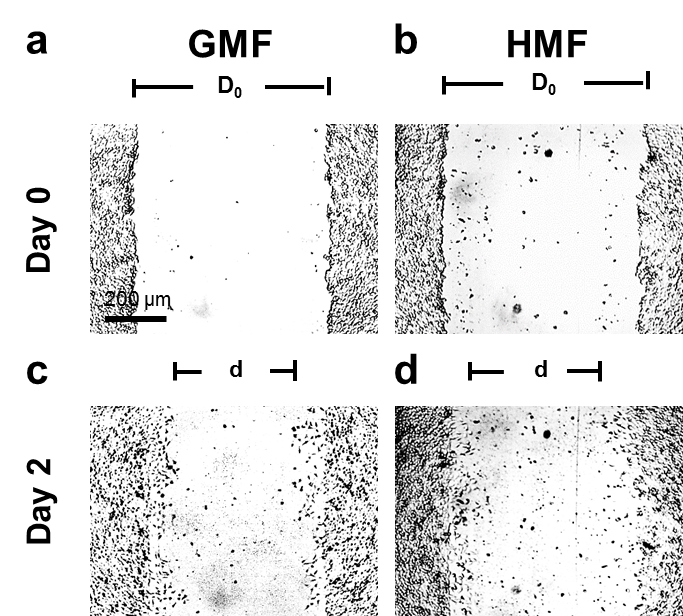
**

**Supplementary Figure S1**. **Reduced Cell Migration in the HMF-exposed SH-SY5Y Cells.** Cell migration was measured by wound healing assay. Cells were seeded into a 6-well plate in DMEM with 10% FBS and grown to a monolayer (complete confluence). Wound lesion was made in each well by a yellow pipette tip. Cells were then incubated in DMEM with 0.5% BSA and transferred to the GMF (a, c) or HMF (b, d) condition for 2 days.


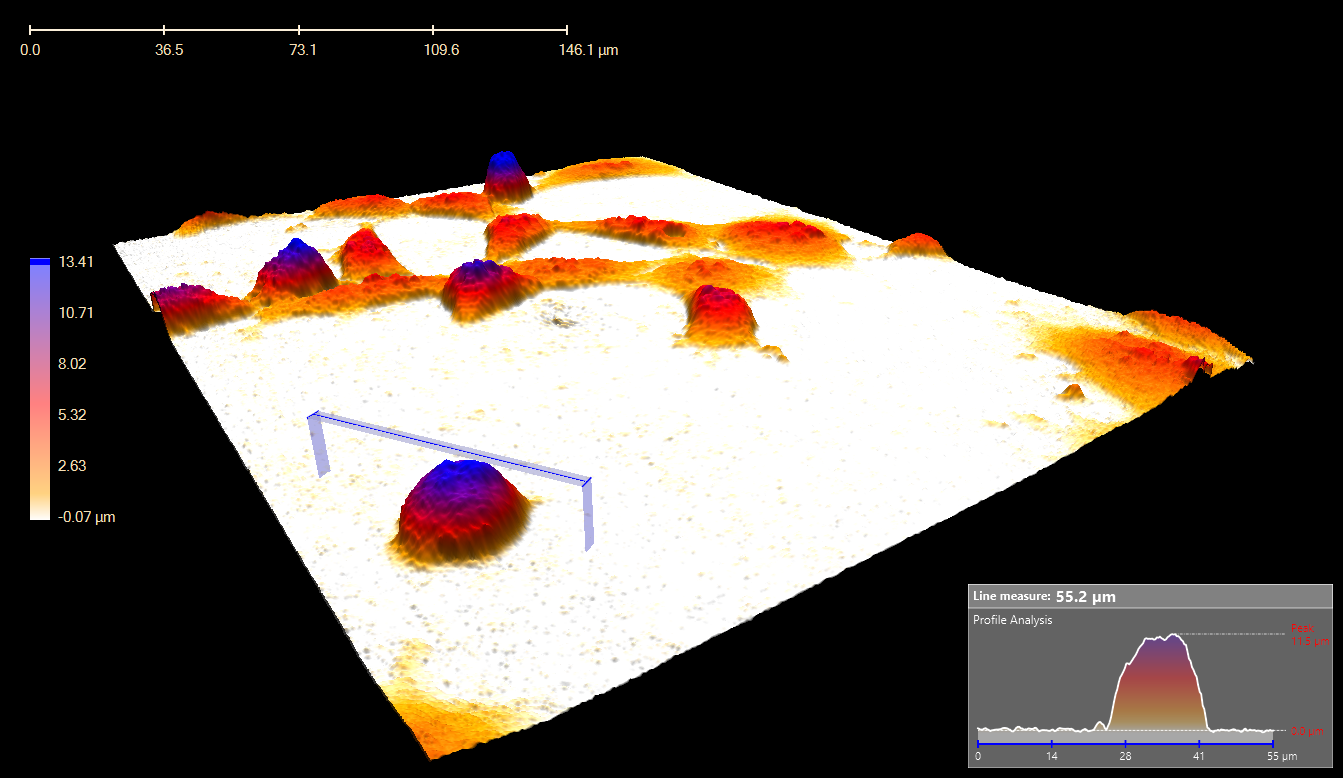


**Supplementary Figure S2. Representative pictures of digital holographic imaging.**

**
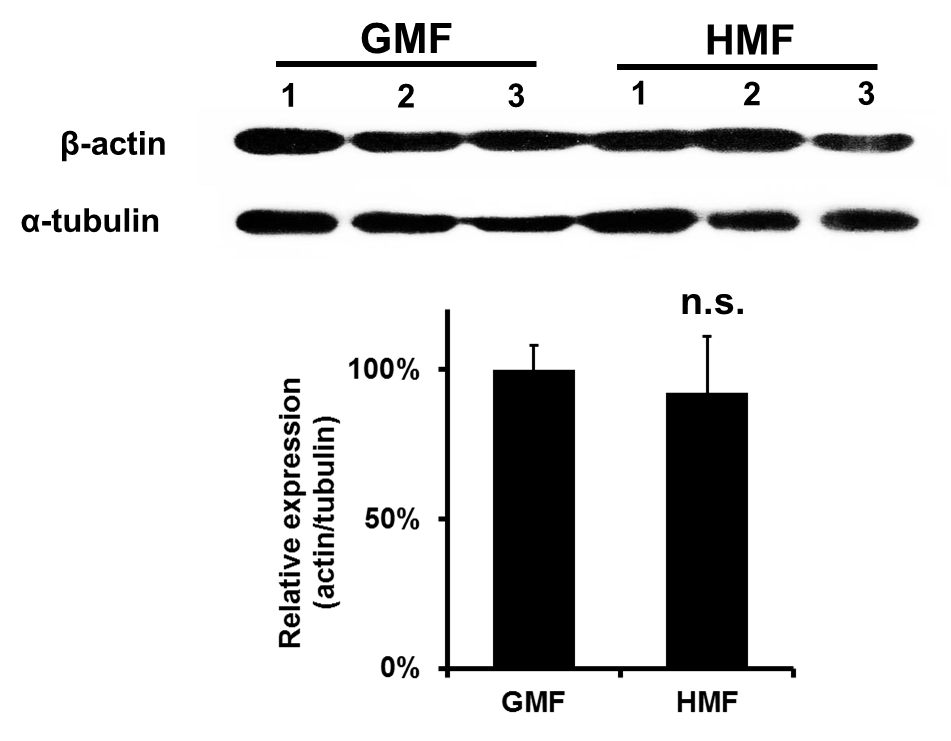
**

**Supplementary Figure S3. Expression of β-actin in HMF-exposed SH-SY5Y Cells.** Western blotting on protein samples of 2 day HMF-exposed and GMF control cells showed that the relative expression of β-actin in the HMF-exposed cells was the same with the GMF control. α-tubulin was the reference control. Three independent experiments were conducted. Data were shown as means ± s.d. The *p* values were calculated with student’s t-test. *n.s., P>0.05*.


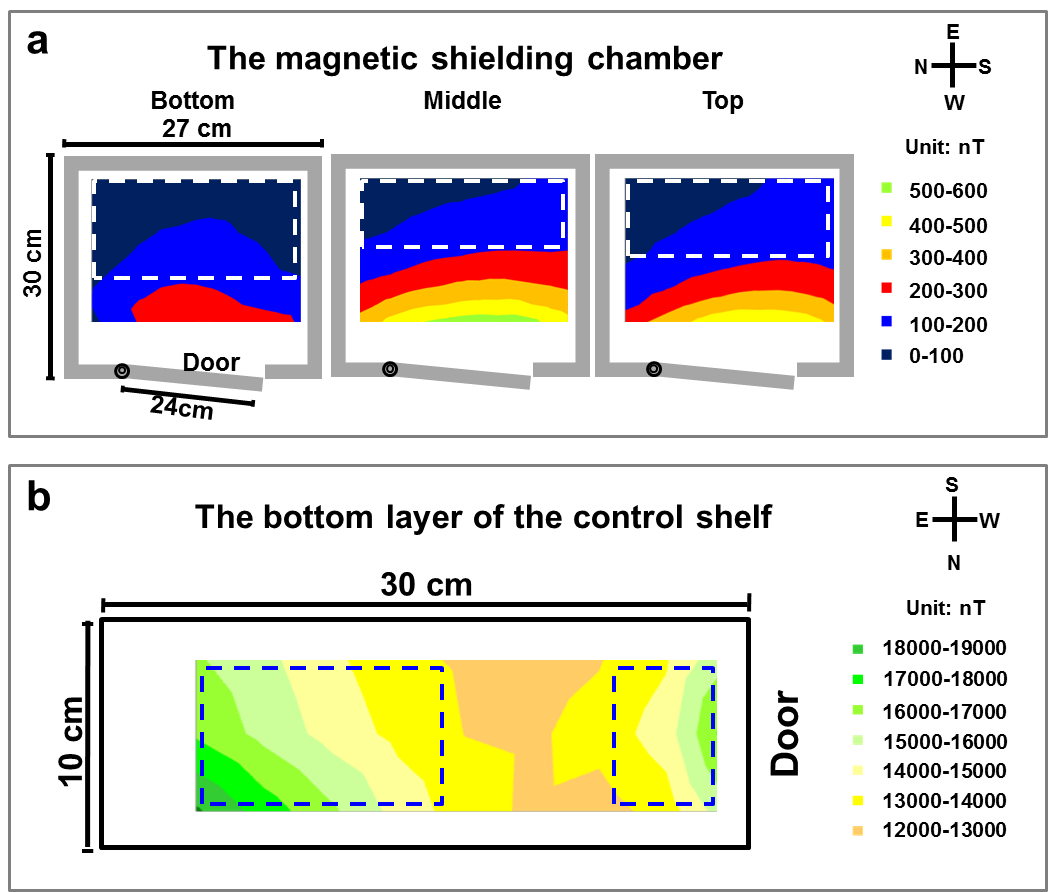


**Supplementary Figure S4. The Magnetic Field Conditions for Cell Culture.** The distributions of the static magnetic fields (SMFs) in the magnetic shielding box (a) and the bottom layer of the control shelf (b) were plotted according to the vector sum of the SMF measurements. The HMF exposed cells were incubated at places with residue SMF lower than 200 nT (The white dashed rectangles). The control cells were incubated at places with SMFs higher than 13 μT (The blue dashed rectangles) (Mo, et al., 2013, Plos One).

**Supplementary Figure S5. The residue SMF at the Center of the Helmholtz Coils System.** The residue SMF at the center of the 40 cm diameter 3-axis Helmholtz coils system was compensated to < 500 nT when the system started working at the “HMF” condition. The HMF field was recorded every 2 min under a non-feedback controlling mode. The vector sum of the SMF measurements was stabilized at 470.0 ± 13.7 nT during the 6 h recording period.


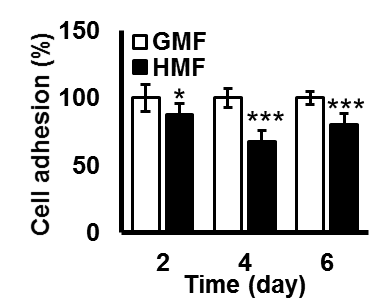


**Supplementary Figure S6. Cell adhesion assay at day 2, day 4 and day 6.** The cell adhesive capacity of SH-SY5Y cells significantly reduced after 2 day, 4 day and 6 day HMF exposure. However, in consideration of the pro-proliferation effect of the HMF, more G2/M phase cells, which is of weaker adhesion, will present in the “even day” HMF cultures under a 2-day interval passaging protocol. To avoid the interference of the pro-proliferation effect of the HMF, only odd day results were plotted and presented in the main text. (Mo et al., 2013).


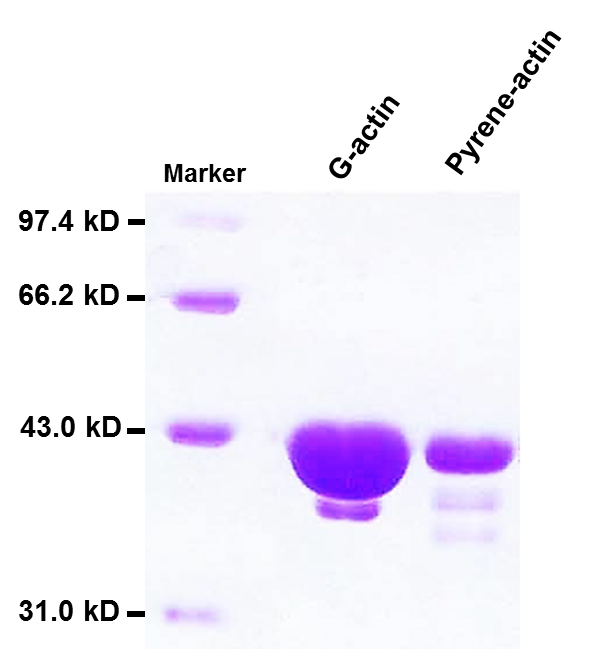


**Supplementary Figure S7. Purification and labeling of G-actin.** G-actin was purified from rabbit muscle. Purified G-actin was conjugated with fluorescence probe pyrene. Prepared pyrene-actin samples were displayed by SDS-PAGE.

**Methods for Supplementary Figure S7.**

**Purification of G-actin**

In brief, rabbit hind-leg skeletal muscle tissue was centrifuged at 4,000 rpm (4°C, 10 min) after homogenization. After discarding the supernatant, the deposit was washed by ddH_2_O for 10 times (4,000 g, 4°C, 10 min) and followed with acetone (Ameresco, Solon, OH, USA) washing for 5 times (5,000 g, 25°C, 10 min). The deposit was lyophilized overnight. The deposit powder from acetone extraction was solved in buffer B (2 mM Tris-HCl, 0.2 mM ATP, 0.5 mM [β-Mercaptoethanol](app:ds:β-Mercaptoethanol), 0.2 mM, CaCl_2_, 0.005% NaN_3_, pH=8.0) at 4°C by gentle stirring for 30 min and collect the supernatant after 3,000 rpm centrifugation (4°C, 10 min). Repeat the dissolving step for the deposit and mix the supernatants from the two dissolving steps. Centrifuge the supernatant at 10,000 rpm (4°C, 1 h) to remove the acetone. Add KCl and MgCl_2_ solutions to the supernatant to the final concentration of 50 mM and 2 mM, respectively. Keep the solutions still at RT for 30 min. Transfer the solution to 4°C and gradually add KCl to the final concentration of 0.6 M by gentle stirring for 30 min. Collect the solution and centrifuge it at 80,000 g (4°C, 3 h). Dissolve the deposit in buffer B and incubate the solution at 4°C overnight. After 3 days dialyzation in buffer B, centrifuge the solution at 80,000 g (4°C, 3 h). The supernatant was the purified G-actin solution. The protein concentrations were determined with a bicinchoninic acid (BCA) protein assay kit (Pierce, Rockford, IL, USA).

**Labeling of G-actin**

Pyrene was mixed with G-actin at molar ration 10:1. The KCl and MgCl_2_ concentrations in the mixture were adjusted to 50 mM and 2 mM, respectively. The mixture was incubated at 30°C for 1 h. Harvest the pyrene labeled G-actin as described above. All the solutions used in pyrene conjugation were wrapped in tinfoils in order to avoid light exposure. The purified G-actin and the pyrene-actin were displayed by SDS-PAGE (SI Figure 7).
